# Extracts and Abstracts

**Published:** 1888-05

**Authors:** 


### EXTRACTS AND ABSTRACTS.

### STUDIES ON THE ETIOLOGY OF CARCINOMA.

[Berliner Klinische Wochenshcrift, No. 10]

[Translated by Dr. Lester Curtis.]

Our present time may be well called the etiological era of medicine. Following the movement in this direction there is a disposition to regard carcinoma as an infectious disease, in the sense that a pathological irritant is deposited in the tissues, which excites there histologically distinct characteristic carcinomatous growths.

The grounds for this opinion, which I have not found collected in the text-books and monographs, I may give briefly as follows :

In the first place, the spreading of the carcinomatous growth agrees throughout, tolerably exactly, with that of an inflammation. Both extend into the lymphatic vessels, and just as we know pyæmic metastases, in the same way are there carcinomatous metastases.

As a second reason, I might bring forward the quite rare cases of general miliary carcinoma, which run their course attended by fever, disturbances of the digestion and circulation, and show the greatest resemblance to miliary tuberculosis, so that the differential diagnosis is only to be made by the use of the microscope.

These cases can be explained, according to our general pathological knowledge, in no other way than by the view that the germs of the disease come into the blood in great numbers, and are deposited in different parts of the body at the same time. The difference which these multiple emboli present to us, from those embolic plugs which the fundamental work of Virchow and Conheim have made known, lies in this, that the surrounding tissue takes on a similar growth. When we consider that the carcinomatous metastases and the nodules in miliary carcinoma occur through an embolic deposition of the cancer germs, since it can scarcely occur otherwise, then the cancer germ, or cancer poison, must, of course, be smaller than the section of the lung capillary, since it must have passed through these capillaries.

As the last reason, may be mentioned tar, paraffine, and chimney-sweep's cancer, described principally by Von Volkmann, in a correct estimation of their etiological relations. These cancers, as is well known, develop occasionally in strong young mechanics, on places of the body where otherwise cancer is a rarity, namely, on the upper and lower extremities and on the scrotum, after a preceding chronic dermatitis. The skin affection heals when the laborers give up their injurious occupation, but in other cases becomes a chronic hyperplastic process, which often effects the whole body and develops sometimes an eruption like psoriasis, or even papillary excrescences. Sometimes the character of this skin inflammation changes and becomes of a decidedly malignant carcinomatous character.

We must, of necessity, grant that some sort of influence, standing in a certain relation to the tar and paraffine, reaches the place from without (whether a poison or a living form) so as to cause the benign inflammation to pass into a carcinomatous one, or, histologically expressed, in order that the hyperplastic process may be stirred up to an atypical destructive growth. We may not accept the view that this influence, this unknown agent, forms itself, as it were, first from within outwards in the chemically altered cells; on the coutrary, this comes from without; in short, the agent depends upon an infection.

Other reasons which are usually alleged for the infectiousness of cancer appear not to be so demonstrable and clear.

I might, in addition, enumerate the seborrhagiac skin carcinomas, the cancers occurring after the so-called "psoriasis buccalis" (Volkman-Schuschardt). Also, those carcinomas which arise, as it were, through contact. For instance, Von Bergmann presented briefly to the Berlin Medical Society the case of a man with a carcinoma of the upper lip, which had formed directly over one already existing on the under lip.

Cases also have been described (Israel and others) in which, for example, after a carcinoma of the tongue or of the buccal cavity, secondary nodules had grown in the digestive tract; and cases are reported in which, after tapping a carcinomatous peritonitis, the growth had followed along the line of puncture. And finally reference is made to carcinomas occurring after chronic ulcers of the leg, bone fistulas, tooth fistulas, burns, etc.

Though infection can not be denied in all these disturbances, still the cases are not absolutely demonstrative, for the disease could also have occurred in these places without preceding inflammation; it is also quite natural that in a vigorously growing carcinoma, the growth should follow in the direction where the least mechanical resistance exists, *i. e.*, in the line of puncture.

Whether the above reasons justify the view of the infectiousness of carcinoma must be left to the judgment and convictions of each

But we can, on the foundation of histological and bacteriological studies, assert with tolerable certainty that the irritant of carcinoma can be no bacterium with the properties which we have become acqainted with in the bacteria. All the pathogenic bacteria known to us, planted on any part of the body, cause disturbance and processes of homoöplastic or homologous nature that is, disturbances and processes which are formed and kept up by growths and proliferations of the cells which are already in the place.

Tubercle, for instance, is nothing more than a collection of round cells and epithelioid cells, whose histogenesis has not been observed to be perfectly uniform.

Great actinomycotic tumors, also, looked at histologically, are essentially only rankly growing connective tissue and white blood corpuscles. Connective tissue, white blood corpuscles, and fixed cells exist everywhere in the body. These processes are, therefore, homoöplastic or homologous.

Up to this time we know no bacterium which can induce heteroplastic growths, *i. e.*, growths of such cells, or such typical compounds of cells as are not at all present in the infected spot concerned. But now, as is well known, the metastases of the carcinomas and their secondary nodules are mostly of the same histological epithelial structure as the primary cancer, even in the place where no epithelium is usually present. Every carcinoma of the mamma, for example, causes in the axillary glands the typical epethelial nests, although, no epithelium is present in the lymphatic glands.

In this connection, after a hard cancer (Horn-Krebs) of the skin, there resulted in the neighboring lymph glands, not only a typical carcinomatous epithelial growth, but even the well-known corneus structure was present exactly as in the primary skin cancer.

This typical continued repetition of an epithelial growth in a place where no epithelium at all occurs, we cannot explain by a simple bacterium. If we suppose there is a micro-organism or a disease-irritant, to which we allow a certain relation to the cell, or, in the sense of our kariokinetic studies, to the cell nucleus or the divisions of the nucleus, so that the micro-organism together with the cell, or the infected cell, affords the first foundation for the further infection, the theory would much rather harmonize with the facts. We could very well imagine that the infected nucleus or division of a necleus might easily pass the pulmonary capillaries and set up heteroplastic growths. In any case the mechanism of the etiology of carcinoma is much more complicated than many authors seem to imagine; and to-day it is much more difficult to explain fully the etiology of carcinoma than it was to make clear the etiology of tuberculosis at the time of Conheim and Salomonsen.

Up to this time, so far as I have been able to look over the literature, no investigator appears to have made a successful inoculation of a cancer from one animal to another, or from a man to an animal, or from one man to another, or upon the same individual.

The earlier investigations were all spoiled, since circumstances were not such as to avoid suppuration and pyæmia; accordingly that was looked at as general inoculated carcinoma, which was in reality a focus of embolic pyæmia.

The one case of Langenbeck, also, which would be gladly brought forward as one of successful inoculation, appears not to be raised above this doubt. For, not to mention that it occurred at a time of the greatest confusion in regard to the carcinoma question, when men like Virchow, Müller, Henle, Reichert, Remak, and others, were busied in making firm the histological character of carcinoma, it might be a matter of great difficulty to make a correct diagnosis out of a nodule the size of a lentil in the lung of a dog.

Virchow, himself, also, in his great work on tumors, says that, judging by the forms, cancers resemble more the growths already present in the dog than such as are produced by inoculation with human cancer cells.

As to the cases of Nowincki it seems only necessary to mention the circumstance that only a preliminary communication has appeared, but never, so far as I know, a completed work.

I, myself, therefore undertook, in the laboratory of Professor Weigert, investigations, aseptically, with absolute freedom from suppuration, on mice, rabbits, and dogs. I inoculated under aseptic cautions, subcutaneously, among the muscles or in the abdominal cavity. Since the inoculations ran, on the whole, a tolerably similar course, a description of one will suffice for a record.

On the 14th of November pieces of carcinoma of the size of a small lentil were inserted under the skin of the back of four white mice, two hours after extirpation (mammary carcinoma). For this purpose the hair was cut off from the place of inoculation; it was washed with sublimate; then this was removed with alcohol, and the alcohol with ether.

November 18. Fixation of the piece on the skin beginning.

December 3. Piece of carcinoma somewhat enlarged, grown firmly to the skin, loosely to the subcutaneous connective tissue where it may be moved. Mouse quite lively.

December 7. Tumor decidedly enlarged, as large as a large bean, feels hard like a carcinoma. Mouse lively.

December 11. Tumor grown still firmer to the skin, appears to have moved upwards. It has become smaller, but is still larger than a lentil.

The tumor was cut out, together with the surrounding skin, and a microscopic investigation undertaken. This gave the following result: Three zones may, without difficulty, be distinguished in a microscopic section.

(a) A homogeneous, structureless centre. In the middle of the cancer node, a part of the cancer mass is not at all present, but there exists a longish oval opening corresponding with the form of the piece of cancer. This opening is evidently the result of softening and absorption of the central piece. Farther towards the periphery, there are found some round cells in the structureless zone.

(b) The second zone is the remainder of the inoculated and now entirely changed piece of cancer. Of the alveolæ furnished with epithelium, there is scarcely anything to be seen, only on some places the former cancer structure is feebly indicated and recognized with difficulty. On the other hand, the whole piece of cancer is filled with numbers of easily stained round cells. Near by can be recognized large epithelial cells stained very feebly or not at all, and spindle cells in exactly the same condition.

The staining is, for the most part, still only possible in the nucleus, while the protoplasm is quite pale and difficult to recognize.

(c) The third zone is separated at the periphery, especially in the sub-cutaneous connective tissue. It consists, there, of a vigorous growth of young, succulent connective tissue with large spindle cells. In the further course these cells become smaller and the connective tissue becomes poorer in nuclei, which keep on growing smaller until they become like the ordinary subcutaneous connective tissue.

The many blood vessels are noticeable. They are filled to distension and bear a wreath of extruded white blood cells in their walls and around them. These vessels enter, for the most part, from the side and from below, *i. e.*, from subcutaneous connective tissue, and evidently send branches into the changed cancerous growth. In many places they are very numerous and run in a radial direction or parallel to one another.

If the natural course of the destiny of the inoculated piece of cancer is not interrupted, then it will be either perfectly absorbed until there remains at last nothing more than a small scar from the inoculation ; or the tumor, after the connection with the rete, becomes smaller and continues to move upward, *i. e.*, toward the surface of the skin. The skin ulcerates at some place and the tumor is, in a few days, pushed out. After this, the tolerably round and smooth bordered wound in the skin soon closes.

That the increase is not to be ascribed to an independent growth of the tumor, which is only successfully combatted by the peculiar resisting power of the animal tissues, or through the constitutional relations of the animal, is shown in this way, that one gets exactly the same picture of adhesion and enlargement, then of diminution, extrusion, or absorption, if he implants physiological tissue in place of the piece of carcinoma.

I have for this purpose made use of a lymphatic gland or a piece of a freshly excised breast gland.

The result of all inoculations I can so far sum up : I did not succeed in animals—mice, rabbits, dogs—in producing a carcinoma artificially by implantation. An enlargment of the piece of carcinoma which has been introduced often occurs, but it is only apparent, and is to be explained by the penetration of numerous white blood corpuscles, the new formed blood vessels and, perhaps, new formed connective tissue rich in nuclei. An independent growth of the implanted carcinomatous cell compound into the neighborhood does not result. On the contrary, the piece of carcinoma is to be considered like an organic dead body in the living organism.

The result of the inoculation is no better if the animals are placed in the most horrible hygienic relations, *i. e.*, they may be allowed to be hungry and wet. For example, the limbs of three inoculated mice sloughed off through the carelessness of the servant. The animals recovered and were quite healthy again, but the piece of carcinoma did not grow at all.

These negative results, which, as I accidentally learned during my lecture, have been also mentioned by other authors (Oscar Israel and Alberts), do not justify us at all in doubting the absence of results from inoculation. There must first be many more experiments conducted by various authors, in somewhat different form. It should be necessary according to my impression :

1. To change the place of inoculation so as to bring the piece of the carcinoma into different organs and neighborhoods, or to make arterial and venous injections, and other similar modifications. Koch, for example, could produce cholera in the duodenum alone.

2. A certain form of cancer, in a histological sense, should be chosen for inoculation, or a cancer in a certain stage of growth, since it might be possible that the poison, virulent in these only, is divested of power in other cancers.

3. The animals, most properly dogs or apes, might, in the sense of Beneke, be placed in the most favorable predisposition.

Alberts has made an attempt, undoubtedly negative, with this end in view.

The most favorable material is in every case an individual already suffering with cancer, then apes, after that, dogs, etc.

Hahn introduced under the skin of a woman suffering with carcinoma of the breast, a piece of her own cancer, and reported that it grew. Virchow was able to reply, very shrewdly and pointedly to this, that the infection is not demonstrated by this growth alone, since, then, all implantations of hair, as well as all Reverdin-Thiersche's transplantations of hair and skin upon an infection, may be included. Unfortunately the communication of Hahn was only very short, but if he could show that the piece of carcinoma not only grew but penetrated into the surrounding tissue, and this stimulated to a new growth, then an important step in advance would have been made.

According to my views of the etiology of cancer, which I have expressed above, I could not hope to go further in the question of infection, on the line of microscopic study of bacteria. Nevertheless, I undertook this task in order to be clear concerning the bacilli, which are, on various sides, asserted to be the cause of cancer, and also to guard by a warning from unpromising studies those investigators about to enter upon this, as I believe, mistaken path. I do this because a whole class of authors have pronounccd in favor of the bacillus described by Scheurlen, some of these ratifying them through their own observations, some even claiming the priority.

I will only briefly mention that Schill asserts the presence of bacilli in cancer, and of filamentous fungi in carcinoma and sarcoma. Concerning their etiological meaning he certainly says nothing.

Then, O. Friere, in Rio and Perrin, later Barnabei and Sanarelli, both in Siena, asserted that they found the cancer bacillus; indeed, the latter claim to have produced cancer artifically in animals by their means.

Finally, Franke of Von Ziemssen's clinic (Münch. Med. Wochenschr. No. 4) has quite recently confirmed in essentials the investigations of Scheurlen; indeed, he goes so far as to assert that he has found the bacterium in the blood of carcinomatous and sarcomatous patients!

I will delay, however, on the work of Scheurlen only, since this seems to me, relatively, the most of all deserving of attention.

According to Scheurlen, a third of the dried preparations made from the cancer juice, should contain spores that ought to stain in the well-known way. I could not satisfy myself of the presence of these spores, but saw only fat drops, which appear exactly like spores. If a soft piece of cancer is vigorously shaken with ether and chloroform in a test-tube, by which the fat is extracted, then we may convince ourselves that the fat drops which are impressing themselves as spores, diminish and disappear. I have seen quite large cells whose protoplasm appeared granular as though filled with many cocci. But these granules were much smaller than the spores.

Besides on theoretical grounds it may be seen that the discovery of spores must be insecure and contradictory. If so many spores are contained in the juice, then these must grow to bacilli, which must be much easier to see than the spores. Now, Scheurlen says that he has found very few bacilli in the juice. In the sections he has seen neither bacilli nor spores. Besides all this, the spores (?) stain remarkably easily, and grow with extraordinary rapidity to bacilli.

A rich material of cultures and inoculation is at my command. * * * * I was able, therefore, to work with ten carcinomas. I did not confine myself to the nutrient soil used by Scheurlen, but at the same time made use of all at our command; gelatine, agar, combinations of the two, coagulated beef and sheep's blood-serum, human ascites fluid, fluid or coagulated, or gelatinized (5–8 per cent.) or, mixed with agar (1–1½ per cent.), potatoes. I have used the ascites fluid agarized when in a fluid state, because, at one time, I failed in a remarkable way to coagulate this even in a steam apparatus at 100° (212°Fahr.). I can not give the reason for this failure to coagulate. I was obliged to carry the fluid for some distance in great cold so that perhaps the albumen was modified.

For coagulating the blood-and-ascites-serum I found the well-known Koch's dry chamber required much time, and was expensive on account of the great amount of gas required. I made use, therefore, of an apparatus which is founded on a water bath, in which only the ends of the tubes lie obliquely in the water. In this is a thermometer on which the temperature is read at which the serum coagulates; for it is proper to determine this temperature of coagulation, since it is not at all constant and by no means fixed at 60° (140° Fahr.). I was often obliged to go to 78°, 80°, 81° (172.4°, 176°, 177.°8 Fahr.). After determination of this temperature, cold water is poured in to 10° (50° Fahr.) or less, a row of tubes is laid in the water-bath apparatus, and the water heated to near the point of coagulation. It is very easy to keep a certain temperature by regulation of the flame or pouring in of water.

By this water-bath chest a number of tubes are prepared in a very short time (10 minutes), while with Koch's chest it is often necessary to wait half an hour and longer, and a temperature from 80° to 85° (176° to 185° Fahr.) is very difficult to obtain. To be sure, the serum is often somewhat less clear.

In the two first carcinomas, I made use, like Scheurlen, of cancer juice; in the following inoculations, always of pieces of cancer which, now and then for purposes of demonstration, were as large as a cherry. For the most part I have inoculated.

10 tubes gelatine.

15 tubes agar.

20 tubes beef and sheep's blood.

20 tubes human ascites fluid.

The latter was usually coagulated, but with two carcinomas, quite fluid human serum was used, agarized and gelatinized, since this would not coagulate. With the exception of the gelatine, the tubes were put in a thermostat at a temperature of 39° (102.2° Fahr.), and remained there at least one month, very many for more than two months.

Although the inoculations undertaken by me are more than 350, a specific bacterium has never grown, so that I am justified in the following proposition:

We do not succeed, with all our present culture soils and usual methods, in cultivating a bacterium from a cancer which stands in an etiological relation with the growth.

Of course, I have sometimes got bacteria, as is not to be avoided in the cleanest and most skillful work in bacteriology; there were, on an average, among twenty tubes always two or three contaminated, therefore a very good result. Among others I have got a yellow sarcina, and once, the *Staphylococcus aureus* from a carcinoma that I carefully inoculated the second time, twenty-four hours after extirpation, while the inoculation of the same cancer the first time did not give this purulent bacterium. It must therefore be granted that this bacterium, the *Staphylococcus pyogeneus aureus*, existed in the air of the laboratory.

But Scheurlen's bacterium I have never obtained, except in one inoculation, considered below, and can assert that it was not contained in the carcinoma.

Since Scheurlen not only holds firmly to his baccillus, but asserted that he had verified the diagnosis of cancer by a puncture for the gastric juice and the discovery of the bacillus, therefore, I do not consider it snperfluous to remark that I have made a subcutaneous injection of the bacilli given me by Scheurlen himself, in dogs, rabbits, mice, and a dove, without producing in any way a pathological disturbance.

I will not enter further upon a wider criticism of the work itself, only I might emphasize that if so many spores are contained in the cancer juice, Scheurlen, himself, by his own inoculations ought to have obtained much more successful cultures.

Further, it is without an analogy that a bacterium taken out of the body should grow on a single soil only, but afterwards may be transferred from this soil to every possible one, with vigorous growth. Although such a possibility may not be completely denied, Scheurlen certainly ought to have given much greater attention and deeper study to this remarkable fact. He should, at least, have sought an explanation for this discord.

And, finally, a chief characteristic of a bacterium is lacking in the work, namely, the behavior upon agar and gelatine plates.

In fact, on the ground of my inoculations and the above theoretical considerations, I consider every discussion as to the causation of cancer by a bacterium as useless; but, for a total clearing up of the matter, it would still be desirable to investigate what sort of a bacterium the one described by Scheurlen might be, and whether it is often to be found in cancer, naturally, as an accompaniment. Now, it is well known that it is very difficult to fix the identity of a bacterium. Both Koch and his pupils, Carl Frankel, Eisenberg, and others, still hesitate to declare the bacilli of chicken-cholera and the septicæmia of rabbits as identical, although they agree in every respect.

I once found the same bacterium which Scheurlen has described when I placed a piece of carcinoma directly upon a potato. After twenty-four hours at 39° (102. 2° Fahr.), a vigorous growth followed. Comparative investigations of this bacterium with Scheurlen's and that taken direct from the potato without inoculation with carcinoma, has led me to the result that the bacillus described by Scheurlen is to be designated as potato-bacillus. The spores of this bacillus are tolerably resisting. I have, for instance, left a potato in sublimate (1 to 1000) for two hours, then boiled it for two and a half hours in a steam apparatus, and still found the bacillus upon the potato.

I might here refer to an important work by Globig, in which he shows that many bacteria grow for the first time between 60° and 70° (140° and 158° Fahr.), and that the sterilization of blood serum is extraordinarily difficult. Hence it may be seen how easily error may slip in if a series of tubes are not free from germs.

Concerning the point "potato-bacillus," I must make one more remark. These bacilli may be designated, without being very exact as those "which grow easily upon slices of potato and are often observed as settlers upon them" (Flügge, Die Mikro-organismen). Hence the domain of this bacillus is tolerably large. Flügge specifies only four potato-bacilli. But there are many more, although they have not been sufficiently studied, separated, and, above all, named.

I have, as mentioned above, found one agreeing perfectly with Scheurlen's, and enumerate it among the potato-bacilli for the following reasons: In the first place, the morphological relations and the spore formation are exactly alike; then the bacillus is movable; it liquifies gelatine and forms a white skin upon it; then it shows, for the most part, slight, wavy, wrinkled, mesentery-like growths; finally, it is not pathogenic. Every potato-bacillus, *par excellence* (the *B. mesent. vulgar*, Flügge), occurring in earth, dust, decomposing fluids, fæces, etc., possesses all these peculiarities, but, examined carefully, it is not quite identical with Scheurlen's.

The description of the universally distributed *B. mesent. fuscus* (Flügge) fits this much better, only this grows dirty-brown upon the potato, while Scheurlen's grows more reddish, rusty-colored, although this red, on many potatoes, passes into red-brown and brownish, as I have convinced myself.

In case the Schizomycete which has engaged our attention, should still have no name—it has often been seen before—then, perhaps, the designation *B. mesentericus robiginosus* (rustcolored) would apply. But in no case would the name cancer-bacillus, be justified, not even pseudo cancer-bacillus; and for this reason I have given the whole account of the potato-bacillus.

The results of my investigations so far as they had in view the peculiar irritant of cancer, have turned out entirely negative; also, according to the theoretical considerations stated above, it is not to be supposed that this great aim—the discovery of the etiology of cancer—will be attained in a short time. On the contrary, it may here be emphasized, in conclusion, that first of all, the foundation must be laid for an earnest, intelligent, *bacteriological* labor for the etiology of cancer, *i. e.*, the inoculability of the same. Without this foundation, the discovery of a bacterium through culture seems to me not only difficult but useless.

We must not forget that the mechanism of the etiology of carcinoma is in any case very complicated, and that we shall determine this bacillus only after accomplishing successful inoculation with pieces of cancer, and by new methods of cultivation which shall agree with the physiological conditions of the hypothetical micro-organism, or, generally expressed, shall fit in with this mechanism.

### THE PRESENCE OF BACILLI IN SYPHILIS.

[Abstracted and Translated from the German by Dr. Elbert Wing.]

In an Inaugural Dissertation presented at the University of Berlin, Dr. John A. Fordyce, of Chicago, in addition to a review of the literature of the subject, reported some original work of his own. The examinations, made at the clinic for skin diseases of Dr. Lassar, of Berlin, were reported as follows:

CASE I.—Moist papule of the lip. Infection of two months' standing. Treatment, four bichloride injections. Examinations of cover-glass preparations according to Lustgarten's method were negative. When the preparations were not completely decolorized, various cocci, and short, thick baccilli, such as are present in the normal secretions of the mouth, were found.

CASE II.—Late syphilitic ulcer of the scrotum. History was negative as to syphilis. Cover-glass preparations stained according to Giacomi with anilin-oil fuchsin, decolorized with chloride of iron, gave diplococci in groups of three and five, also single ones, double the size of gonococci, which they otherwise resemble. Negative result with Lustgarten's method.

CASE III. — Broad condyloma of the vulva. Inguinal glands enlarged. No treatment. Examinations with Giacomi's method gave bacilli which morphologically correspond with those of Lustgarten, and others half as long and as thick again. Lustgarten's method showed the characteristic slender bacilli, but not in such great numbers as that of Giacomi.

CASE IV.—Papillo-serpiginous syphilide of the arm. Secretion obtained by abrasion of the surface. Results were negative with the method of Giacomi, and also with that of Lustgarten.

CASE V.—Diagnosis, methods, and results the same as in case IV.

CASE VI.—Mucous plaque of the right tonsil, stained with Giacomi's method gave numerous cocci, and short, thick bacilli. Lustgarten's method gave negative results.

CASE VII.—Broad condyloma of the vulva. Inguinal glands were enlarged. Treatment was continued one week. The secretion was stained according to Doutrelepont's method, double staining of saffranin. The bacilli corresponded to the syphilis-bacillus, and also the short, thick ones described in Case VI.

CASE IX. — Syphilitic pimphigus in a child. Double saffranin staining, according to the method of Doutrelepont and Schütz, gave cocci, single, and in groups of two, three, and four; also diplococci resembling gonococci, but larger, and always alone.

CASE X.—Ulcer on the perineum, one month old. Diagnosis was uncertain, but three years prior the patient had an ulcer on the penis, skin eruption, alopecia, etc. The secretion was stained by the methods of Doutrelepont and Schütz. In every field groups of ten to twenty of Lustgarten's bacilli, of crossed and curved forms, as if button-like swellings were found; also cocci, single and in twos and fours, like streptococci, and short, thick bacilli. Both the 1 to 16 acid decolorizing solution of

Doutrelepont and Schütz, and also a stronger, 1 to 8, were repeatedly used without any change in number or variety of forms of the bacilli observed.

Case XI.—Broad condyloma from the anus of a child. Every field was full of cocci in groups and chains, as well as the Lustgarten bacilli, and the short and thick ones mentioned before.

Case XII.—Secretion from a mucous plaque of the tonsil. The method of Doutrelepont showed cocci in great numbers, also bacilli resembling those of Lustgarten—long, thin, crossed, and curved. The cocci were in groups of two to four and in chains.

Case XIII.—Broad condyloma from between the vulva and the inner surface of the thigh. The method of Lustgarten gave an exquisite picture of the syphilis bacilli. Every field contained one or more groups, and every group fifteen to twenty bacilli, which lay partly parallel, partly crossed, and all possessing the characteristics which Lustgarten has described. Doutrelepont's method showed the same bacilli, with others, and also cocci. Staining with Ziehl's solution and decolorizing with 33⅓ per cent. hydrochloric acid, double staining with methyl-blue showed the bacilli, but in smaller number.

Case XIV.—Broad condyloma of the vulva. Roseola syphilitica. There was no treatment. Lustgarten's method of staining the secretion gave a result similar to Case XIII. Doutrelepont staining showed, in addition to the syphilitic bacilli, the other bacilli and cocci in great numbers.

Case XV.—Mucous plaque of the lip. Lustgarten staining of the secretion gave negative results.

Case XVI.—Broad condyloma of the vulva. Enlargement of the inguinal glands; roseola syphilitica. There was no treatment. Doutrelepont staining of the secretion shows syphilitic bacilli in straight and curved forms, with and without the button-like swelling, lying partly singly and partly in groups. Also numerous cocci and various forms of bacilli. Lustgarten's method shows only the bacilli of syphilis. Giacomi's method shows diplococci and streptococci, which resemble gonococci in size and form, but which are not confined in pus-corpuscles.

Case XVII.—Secretion was obtained by abrasion from a small syphilitic papule oı the skin. The Doutrelepont and Lustgarten methods gave negative results. He says: "At this time my attention was called by Dr. Lassar, to a work of Kühne in Wiesbaden, entitled, 'Concerning a universal procedure for demonstrating the schizomycytes in animal tissues,' in which he claims that by the use of two dye-stuffs—crystal-violet and methyl-blue—he can stain a greater number of various schizomycytes in the tissues than by any other means. The method is as follows. Into a dish containing a 1 per cent. aqueous solution of carbonate of ammonium filter a concentrated aqueous solution of crystal-violet until a drop placed upon blotting paper makes an intense, dark-violet speck. Place the dehydrated sections in this fluid for five or ten minutes—if tubercle bacilli are suspected, for one hour—then wash in water and place for one or two minutes in the usual solution of iodide of potassium (Iodine, 1; iodide of potassium, 2; distilled water, 300—Ed.), wash again in water and place in concentrated alcoholic solution of fluoresceine until they are nearly decolorized. The balance of the staining material is then taken out in two dishes of absolute alcohol. Oil of cloves may be used to test the decolorizing. When placed in this oil, if the sections show no more violet clouds the oil is removed with turpentine or thyme, and they are placed in xylol."

Case XVIII.—Broad condyloma between the vulva and thigh. The secretion, stained by Giacomi's method, shows large numbers of syphilitic bacilli. With a Zeiss achromatic objective of 2 mm. focus and ocular No. 8, parts of the bacilli showed unstained, the swelling on the ends being more intensely stained.

In addition, diplococci, resembling morphologically gonococci, were present in groups of ten to thirty, inclosed in pus-corpuscles. These were very numerous and presented the appearance of stained gonorrhœal pus. Besides these there were other bacilli and cocci. Staining the secretion with Kühne's method gave a similar result, the diplococci taking an intense black-blue color.

In six other cases the secretion from hard chancres of the prepuce was examined by both the method of Lustgarten and by that of Kühne. The former showed the bacilli of syphilis, the latter cocci, single and in chains and groups. The cover-glass preparations in the Kühne method were left in the solution from twenty to sixty minutes. The secretion from three hard chancres of the prepuce was examined by Giacomi's method and showed the bacilli.

The examination of the secretion of a hard chancre of the lip, by both Lustgarten's and Kühne's method, was negative.

In two cases of macular syphilitic eruption, the examination of the secretion of papules of the skin gave negative results.

In two cases of late serpiginous ulceration of the soft palate, the secretion was stained in the crystal-violet solution for twelve hours without showing the characteristic bacilli. There were, however, numerous zoöglear masses, styphylococci, streptococci, and bacilli of various size and form. Lustgarten's method decolorized these masses much better but not entirely.

The examination of a mucous plaque of the tonsil gave a similar result.

The examination, by Lustgarten's method, of an ulcerating gumma of the nose, gave a negative result.

These examinations of the syphilitic secretion gave positive results in fifteen cases, and, with one doubtful exception, these were limited to secretions from the genitals or their immediate vicinity. "I can confirm the observation of Matterstock, viz., that the bacilli are more numerous in the secretion from places where the body temperature is constantly maintained, and in which the secretions stagnate and partly decompose. In cases where the hard chancre lies free, and in which the secretions are slight, the bacilli are far less numerous. In cases of hard chancres hidden between the folds of the prepuce, and in those in which the secretion came from condylomata, the micro-oganisms were exceedingly numerous."

A comparison of the several methods of staining shows that while all of them stain the characteristic bacilli, the decolorizing method of Lustgarten most successfully decolorizes the cocci and all other bacilli.

The bacilli of Lustgarten are found in very great numbers both in the smegma preputialis and in the corresponding secretion of the female genitals in non-syphilitic persons. And there is no manifest difference in the power of resistance against alcohol between these bacilli and those found in the syphilitic secretions.

In some cases they withstood the action of alcohol for two minutes after they were decolorized in permanganate of potassium solution and sulphuric acid, but in others not so long.

The method of Doutrelepont (48 hours in a 1 per cent. methyl-violet solution) stains the bacilli of syphilis, as well as others of different shape and size, and also cocci, streptococci, and zoöglear masses. The appearance resembles the one obtained in staining the secretion of broad condylomata by the same method.

In regard to the differential diagnosis between the bacilli of smegma, or syphilis, and those of tuberculosis, the latter, beyond question, have a much greater power of resistance against the action of acids.

The staining by Ziehl's solution, and decolorizing in 33 per cent. hydrochloric acid, frequently gives a negative result with smegma and condyloma secretion. In other cases bacilli appear but sparingly, and disappear upon longer action of the acid.

The finding of bacilli in the mucous plaques of the mouth is to be taken with caution, since there are so many varieties of bacilli in the normal secretion of the mouth, and an error may arise because the decolorizing methods do not suffice to differentiate, with absolute certainty, the normal from the pathological.

In the tissue of five freshly excised hard chancres of the prepuce, stained according to both Lustgarten's and Doutrelepont's methods, bacilli were found in two cases. Of thirty sections of each chancre, six in all contained bacilli. Two sections contained twelve bacilli, singly and in groups of from two to four; in one case crossed, in another parallel. They were situated in cells, and of the size and shape described by Lustgarten, and were located as a rule at the border between sound and infiltrated tissue. In some sections bacilli were found upon the surface of the ulceration which were not morphologically distinguishable, except that they did not lie in the cells.

The very great infrequency of the bacilli renders the search for them exceedingly painstaking, and when they are once found it is no easy matter to again bring them into the field of vision. Indeed, it is possible only by the use of a mechanical stage and a Maltwood finder, or its substitute.

In three hard chancres examined, most careful search failed to reveal any trace of micro-organisms. However, with sections from two of these chancres, which lay in the crystal-violet solution twelve hours, he had better results. In every two or three of these sections bacilli appeared which were in every way identical with Lustgarten's bacilli. They were stained an intense blue-black, were often in the cells, and lay at the border of the infiltrated tissue. In one polyonal-formed cell two bacilli were crossed, in another two lay parallel, and in still another they were disposed singly.

The largest number found in any one section was twelve. In addition, groups of cocci were found both upon the surface of the chancre and in the infiltrated tissue. Every section contained one or two groups of cocci of from fifteen to twenty, whose size and shape indicated the cocci of erysipelas.

The most satisfactory results were obtained from a recently excised condyloma of the labia majora, stained according to the method of Kühne. Every section contained groups of cocci, both upon the surface and in the infiltrated tissue, which resemble those mentioned above. Every second section contained bacilli of syphilis, singly and grouped. In one section he was so fortunate as to find one collection of twenty such bacilli in the subcutaneous cell-infiltration; they lay crossed, parallel, and in every possible angle to one another; they were 3.5 to 7 $\mu$., and occasionally joined themselves in strings of three or four. Their form presented the greatest variety, straight rods and S forms; many exhibited the button-like swelling at the end, and excrescences at the middle of the bacilli were seen, as if a spore were separating from them. He also found similar appearances in bacilli from the secretion of chancres, stained by the same method. Near these groups of bacilli lay cocci, at times in groups of fifteen or twenty, and at times in chains. One of the latter suggested the clumps of nucleoli (Haufen von Körnchen) which Doutrelepont and Matterstock have described as decaying (zerfallene) bacilli. In the cell-infiltration there were many of the mast-cells of Ehrlich, with bright-blue colored granules on their edges, which lay in double rows, somewhat like an inverted V. These occur at moderate intervals, and bear no relation to cell-granulation, but look as if they were bacilli, as they represent the description of Birsch-Hirschfeld. He had himself seen that they do not belong exclusively to syphilitic infiltration, since he had met them also in simple condylomata.

In fifteen sections from simple condylomata (spitzen condylomen) stained with crystal-violet, cocci alone appeared, and they were mostly upon the surface.

The investigation of a hard chancre of the lip by Lustgarten's method gave a negative result.

The small number of his investigations does not permit a conclusion to be drawn as to the relative value of the methods of staining sections of tissue which were employed. However, it seems to him that the method with crystal-violet stains a larger number of the bacilli than either that of Lustgarten or Doutrelepont. It can not be said what the shortest time is in which, by that method, the bacilli can be stained. With cover-glass preparations twenty minutes suffices. Attempts to find bacilli in sections which have been stained for less than twelve hours were not made. It is probable that a shorter time would suffice if a warming-oven were employed. The decolorizing method of Kühne was the onlv one employed.

It is clear that a conclusion in regard to the diagnostic value of the bacilli when found in cover-glass preparations can not be drawn, since they appear in the normal secretions of the male and female genital organs.

The bacilli found in the syphilitic tissue, however, stand related to the pathological process, but what the nature of the relation is can only be determined by further investigation, culture-trials, and inoculation experiments.

### REPORT OF THE ROTUNDA HOSPITAL.

Obstetricians all over the world are always eager to read any report from the Rotunda Hospital, Dublin, and that for the three years ending November, 1886, has just been published in the *Transactions of the Academy of Medicine in Ireland*, Vol. V, 1887, having been presented by Mr. JOHN LILLY LANE, Ex-Assistant Physician to the Obstetric Section of the Academy last June.

For the past three years the corrosive-sublimate solution has been more freely used both for the hands and for intra-uterine injections, and has almost taken tne place of carbolic-acid solution, except in cases of considerable hæmorrhage, suspected renal mischief, or great corpulence; the reason of the continued use of the carbolic-acid solution in these cases being that as absorption is so much greater there is danger of mercurial poisoning manifesting itself by diarrhœa, tenesmus, bloody stools, etc., while with the use of the 1.80 solution of carbolic acid no bad symptoms have been noticed. Iodoform pessaries are not used so frequently now, and only when the lochial discharge becomes fœtid, and continues so after the first washing out of the uterus.

During the three years there were 3,414 patients attended in the hospital, including twelve cases of threatened abortion. These twelve cases are deducted in the following percentages. There were forty-three deaths from various causes, making a mortality of 1.25, or one patient in 79.39. One death occurred from supposed acute yellow atrophy of the liver, about a week after the patient left the hospital, which she did contrary to orders. One death occurred from septicæmia the day after the patient went home, this also contrary to orders. Two patients died over five weeks, one over four weeks, and three over three weeks after delivery; seven deaths were due to hæmorrhage, two to acute yellow atrophy of the liver, three to convulsions, one to exhaustion from discharge from encysted peritonitis, one to congestion of the lungs, three to phthisis, one to tuberculosis of lungs and brain, one to acute bronchitis, three to pleuro-pneumonia, one to pneumonia and Bright's, one to syncope, one to excessive vomiting with hæmatemesis, and eighteen to septicæmia. These eighteen from septic infection give a mortality of .52 per cent., or one in 189.6; but a better criterion of the state of the hospital is the number of patients that had absolvtely normal temperatures and pulses during the puerperal period. Taking the maximum physiological temperature as 100.4° (38°C.), the percentages of cases that did not exceed this maximum at any time during the puerperal state is as follows for the three years: In 1884, 74.66; in 1885, 83.54; in 1886, 85.75.

Not only has there been a steady increase in the number of patients each year, but the general health of the hospital has improved, as may be seen by the following numbers showing the number of abnormal temperatures for each of the three years.

| | 1883-84 | 1884-85 | 1885-86 | Total |
|---|---|---|---|---|
| Number of patients in hospitals each year............ | 1,109 | 1,112 | 1,193 | 3,414 |
| Temperature above 100.4°, not above 102.2° Fahr.......... | 176 | 94 | 93 | 363 |
| Temperature above 102.2°, not above 104° Fahr............ | 86 | 68 | 58 | 212 |
| Temperature above 104°, not above 105.8° Fahr........... | 19 | 21 | 17 | 57 |
| Temperature above 105.8°, not above 107.6° Fahr.......... | .. | .. | 2 | 2 |
| Total abnormal temperature each year................. | 281 | 183 | 170 | 634 |

*Forceps Cases.*—There were 203 forceps cases, a percentage of 5.96, or one in 16.75. Of these, six died, but only two from septicæmia, one having a fœtid discharge when admitted. In the other case the child was dead and œdematous when delivered; placenta adherent, removed, when it, with the discharge, was found to have a very fœtid smell; slight post-partum hæmorrhage. The six cases gave a mortality of 2.95 per cent., or one in 33.83 in forceps cases. The forceps are not applied in the hospital unless there are positive indications to warrant their application either on the part of the mother or the child. Those on the part of the child that are considered as indicating danger to life are a very quick or a very slow fœtal heart, or the escape of meconium *per vaginam* when the head presents. Dr. Neville's axis-traction forceps are those most frequently used. Mr. Lane thinks it has many advantages over the axis-traction forceps with rods, being more portable, more easily applied, distends the perineum less, can be used, if desired, as a simple forceps, can be more easily cleansed, and there is no danger of injuring the vagina by the mucous membrane being caught between the blades and the rod.

*Treatment of Retained Placenta, Especially when Placenta is adherent.*—Mr. Lane divides the subject into two classes: 1. Cases of simple retained placenta. 2. Cases of retained adherent placenta. The first class he divides into two heads: Cases without irregular contraction, and cases of irregular contraction. The treatment of retained placenta without irregular contraction is very simple, provided the bladder be empty; but simple as it is Mr. Lane thinks that the hand is often passed unnecessarily into the uterus to remove it. It is often said that the hand should exert steady pressure on the fundus during the third stage of labor; but if this be not properly done, instead of doing good it will actually do harm, for, as the fundus is occasionally deflected to either side, usually the left, when pressure is made in the mesial line in the hope of expressing the placenta, the later flexion is still more increased thereby, folding the uterus as it were on itself, and pressing the placenta toward the fundus rather than from it through the os. He thinks that the present practice in the hospital, moving the fingers lightly over the uterus, is preferable, and much less tiresome to the hands of the operator. Of simple retained placenta he has seen some cases in which the placenta was immediately expelled when the fundus was raised out of an abnormal position and without pressure.

Cases of irregular or hour-glass contraction are sometimes met with in the third stage, and are said to occasionally occur naturally, but I believe it is much more frequently produced artificially, by the hand being placed during this stage not on the fundus, but somewhat lower down—possibly at the ring of Bandl—and pressure and friction there continually used, exciting and causing the circular fibres situated in that particular part of the uterus to contract tonically. If this contraction-ring be below the edge of the placenta, it will prevent it from getting down into the lower segment of the uterus, or it may be gripped by the ring, in either case, perhaps, necessitating the introduction of the hand for its removal. Cases of retained placenta, due to irregular contraction, may be sometimes overcome, Mr. Lane thinks, by removing the hand from the uterus, and douching it out well, preferably with hot antiseptic solution, but with plain water if the hot solution be not at hand.

When the placenta is adherent, Mr. Lane believes the proper treatment is to pass the hand or the fingers into the uterus and detach it, but he considers that if the operator's hands be not perfectly aseptic, this is the most dangerous operation in midwifery, except Cæsarean section. It is not always possible to keep the hand within the membranes during the operation, owing to the friable nature of the placenta, necessitating the removal of small pieces at a time. In the Rotunda an anæsthethic, usually chloroform, is almost always given, in order that the hand may be passed a second time when there is any doubt whether all the placental tissue has been removed. In the three years of the report 37 cases of adherent placenta were removed, a percentage of 1.08, or one in 91.95. Of these 37 patients six died, a mortality of 16.2. But of the six deaths only two were due to septicæmia.

*Laceration of Perineum.*—When the perineum is lacerated over .75 inch the practice is, having douched out the vagina with antiseptic solution, to suture immediately, either with silk or catgut (continuous suture for the latter). The stitches are inserted deeply so as to bring the whole of the torn surface into apposition, and the results have been very satisfactory. If the torn surfaces be not accurately apposited the lochia would probably collect between the edges of the wound, causing them to become unhealthy, especially if the discharge be fœtid, and this condition, when once produced, is likely to go deeper, possibly invading the whole depth of the perineum, causing the stitches to slough out, and the wound to gape wide open.

*Retention of Membranes.*—In cases of retention of a portion of the membranes the practice in the Rotunda is to make gentle traction, having as a rule tied a ligature on it as close to the vulva as possible, thus gaining a firm hold so that it can be twisted, thus reducing the likelihood of its breaking. "Should the membranes break well inside the vulva, the best course is to allow them to remain there, but the douche may be used, which may possibly cause the piece to come away, and will in any event be beneficial if an antiseptic solution be used. This course is far preferable to introducing the fingers or the hand, whereby air is introduced—a fertile source of fœtid discharge. As to hæmorrhage I do not consider it is ever traceable to the retained membrane, but to the imperfect contraction, which is the very cause of the membrane not being expelled."

*Prophylactic antiseptic treatment of ophthalmia neonatorum* is never adopted until the disease is actually manifest. Both eyes, even though only one shows symptoms, are then treated with nitrate of silver solution, eight grains to one ounce, pieces of lint dipped in cold water being kept constantly on the affected eye or eyes. When only one eye is affected it is well to have the other bandaged up so as to prevent contamination. The number of cases for the three years was .99 per cent., say one in 100, which is very low.

Infantile asphyxia is treated by Schultze's plan. First the finger is passed into the child's mouth, and the mucus removed as far as possible. The child is then placed on its back, and the operator's hands are put under its back so that they lie at each side of the spine, the fingers in the direction of the child's lower extremities, and its head resting, or partially resting, between the ulnar sides of the operator's hands. The index fingers are then passed underneath the axillæ from behind forwards, the remaining fingers continuing to support the back. The operator now stands up, allowing the child to hang with its feet downwards. The child is now swung upwards so as to cause the legs to fall over the body, and the thorax to be compressed by the thumbs, and then after an interval the legs are swung back to the original position, so that the child will be, as it were, in the vertical or standing position, whence it is again hoisted to the second position. This movement is repeated eight or ten times, and then the child is placed in a warm bath for a few minutes, during which time any mucus that has collected in the larynx is removed by aspiration through a catheter. Care should be taken not to jerk the child during the movements, lest some of the viscera be injured.

*The Incubator*, Mr. Lane is satisfied, has been the means of saving the lives of many infants in the hospital, since, notwithstanding the efforts of the mothers to keep them warm, the extremities of the children sometimes become œdematous and frequently assume an almost erysipelatous appearance, feeling quite cold, and accompanied with an inability to draw the breast, and marked fall of temperature; but when these children are placed in the incubator an improvement is visible in twenty-four hours, and generally after another twenty-four hours the child seems completely restored. Such children always get wine-whey.

---

### ILLINOIS STATE BOARD OF HEALTH.

The regular quarterly meeting of the Illinois State Board of Health was held in this city April 19 and 20.

The Secretary presented his quarterly report, in which he says that notwithstanding the exceptional severity and sudden and marked changes of the meteorological conditions which occurred during the quarter which ended March 31, 1888, there has been no serious increase of the mortality rate, as a whole, nor have the diseases which assume an epidemic form prevailed to an unusual extent. The mean temperature for the quarter has been from five to eight degrees lower throughout the State than for any corresponding period during the last 15 or 20 years—the figures varying between these extremes in various localities.

This country cannot be regarded as safe from the menace of cholera so long as, on the one hand, and under existing conditions of maritime quarantine, we continue to receive large numbers of immigrants with their household effects from the recently cholera-infected districts of Europe; and, on the other, so long as the disease continues its ravages on the South American continent.

It is, at least, the part of prudence not to relax vigilance or effort so long as these conditions exist.

Considerable preliminary work has been done toward investigation of the water supplies of the State, in connection with the sanitary survey of the various localities to secure proper collection of the samples for the chemical and biological examinations, and for collecting and utilizing data concerning borings, shafts, and wells sunk in various parts of the State.

### WEAR AND TEAR OF THE MEDICAL PROFESSION.

Since the close of 1887, I have been enabled to partly tabulate and analyze a mass of material accumulated during the past ten years bearing on the wear and tear of the medical profession of Illinois.

Even prior to the beginning of that period, I had become impressed in a general way with a conviction that this wear and tear was underestimated; that the active practice of medicine was not so conducive to longevity as is popularly supposed, nor as writers on such subjects—basing their conclusions on the data obtained from medical biographies, cyclopædias, etc.—had been led to believe.

The source of error in this latter instance is obvious. The subjects of biographies, cyclopædia articles, memoirs, etc., are necessarily the men who have attained eminence, or at least prominence ; and, in the nature of the case, prominence in the medical profession is largely the fruit of long service and length of days. In other words, the exceptional class which, partly by very reason of long life, has attracted most attention has been hitherto taken as an indication of the longevity of the profession as a whole. Thus we find one writer (Dr. Geo. M. Beard) citing the deaths of 490 Massachusetts physicians whose average age at death was 57 years, and 55 out of every 100 of whom attained to 70 years. The average age of the subjects of Gross' Medical Biography was 59 years. although it is ingenuously added that these "included several who died before their prime" Similarly Thacher's Medical Biography makes mention of 145 physicians, and the fact that their average age at death was 62.8 years is quoted—as are the other instances—as proof of the longevity of medical men.

Still another fact should be taken into consideration in the case of the class who figure in biographies. It is composed very largely of city physicians, and of the men who, in the smaller towns, are in a position to select their practice and adjust their labors with some regard to regular hours of sleep, meals, and relaxation. Comfortably housed at home, properly protected from the weather when making visits, free from the harassing cares of the *res angusti domi*, and beyond the torturing anxiety which too often besets the struggle for practice—the conditions of life in these cases are undoubtedly favorable to longevity.

But these are the fortunate few who bear no more numerical relation to the rank and file of the profession than the general officers do to the rank and file of an army.

Compared with these biographical subjects, upon whose length of honorable and successful years is predicated the assertion that the wear and tear of the profession does not prevent its members from attaining a high average longevity—compared with these I have, as the result of an extensive correspondence and systematic record, obtained data which show that the average age at death—in Illinois, at least—is not much over 52 years, and that only about 11, instead of 35, in every 100 attain the scriptural limit of three score-years and ten.

In older communities it is entirely probable that this rate may be exceeded. In Massachusetts, for example, the average age at death of 1,166 physicians, occurring during a period of nearly thirty-two years, is given as about 55 years ; but the Illinois statistics—collected with painstaking care and dealing with more than double the number living annually—do not furnish any such favorable result. To a very great extent the discrepancy between Illinois and Massachusetts is due, no doubt, to the different conditions which obtain in the two communities—the one a comparatively newly-settled State, with a population containing less than the normal proportion of the middle-aged and beyond ; the other one of the oldest settled commonwealths, with an excess of ages beyond the middle life, and with what Dr. Holmes calls the "adjustable conditions of living" so perfected as to materially conduce to the prolongation of life. But in addition to this difference there mnst also be taken into consideration the radical difference in the modes of collecting the data upon which the average age at death has been computed.

For Illinois these data have been obtained through official relations with an aggregate of some 14,000 physicians during a period of over ten years. The personnel may be taken as fairly representative of the profession generally, since it is composed of about one-sixth of physicians of a large city, Chicago, and the remainder of physicians of smaller cities and towns. During these ten years there has been an average of 6,000 living per annum, and the aggregate deaths have been about 800, or an annual death rate of 13.3 per thousand. These round numbers, and the period covered, are cited to show that the data are extensive enough to insure substantially trustworthy results in the tabulations and deductions.

Of the total number of deaths—800—the age at death and the cause of death have been obtained in 686 cases, and from these data, from information contained in the official registers of the State Board of Health, from the statistics of the United States census and the mortality reports of Dr. John S. Billings, the following tables have been constructed.

Table No. 1 shows the annual number of Illinois physicians living at given ages; the annual number of all Illinois males living at the same ages; and the annual deaths for each class at the same ages. The ages are grouped in decades from 30 to 80: under 30 they are comprised in one group, embracing the ages from 24 to 30; and all over 80 form one group. The limits of the first group—24 to 30—are fixed by the fact that no deaths are reported under 24 years of age.

Table No. 2 shows the yearly death-rate per 1,000 among Illinois physicians at the ages specified; among the whole male population of Illinois, embracing all occupations, physicians included, and among both sexes of the whole population of the United States.

TABLE I.—*Average number of physicians in Illinois living annually at grouped ages, and of all males in Illinois at same ages; Average No. of deaths annually of each class*, 1878 *to* 1887, *inclusive.*

| AGES. | LIVING ANNUALLY. | | DEATHS ANNUALLY. | |
|---|---|---|---|---|
| | Physicians. | All Males. | Physicians. | All Males. |
| 24-30 | 1,409 | 156,662 | 9 | 1,033 |
| 30-40 | 1,740 | 201,889 | 13 | 1,536 |
| 40-50 | 1,304 | 144,997 | 15 | 1,501 |
| 50-60 | 877 | 97,536 | 17 | 1,626 |
| 66-70 | 441 | 49,003 | 15 | 1,570 |
| 70-80 | 177 | 19,717 | 9 | 1,269 |
| Over 80 | 42 | 4,542 | 2 | 572 |
| Totals | 6,000 | 674,346 | 80 | 9,107 |

TABLE II.—*Yearly death-rates per* 1,000 *of physicians in Illinois, of all males in Illinois, and of both sexes in the United States—at grouped ages.*

| AGES. | YEARLY DEATH-RATE PER 1,000. | | |
|---|---|---|---|
| | Physicians. | All Males. | Both Sexes. |
| 24-30 | 6.4 | 6.6 | 9.7 |
| 30-40 | 7.4 | 7.6 | 11.9 |
| 40-50 | 11.5 | 10.3 | 13.9 |
| 50-60 | 19.5 | 16.5 | 18.1 |
| 60-70 | 31.1 | 32.0 | 23.8 |
| 70-80 | 53.0 | 64.4 | 46.9 |
| Over 80 | 476.1 | 126.0 | 113.3 |

An examination of the above tables shows that while the death-rate of physicians in Illinois for the first few years after entering upon the practice of medicine is lower than that of all males in Illinois, and greatly less than that of the whole population of the country at large, it increases beyond that of the former class during the decade from 40 to 50, and is greater than that of the latter class in the next decade.

The obvious inference is that physicians, on entering practice form a class of selected lives, since they have an advantage of nearly 3 per cent. as compared with all males at the same ages—that is, from 24 to 40; and of over 50 per cent. as compared with the total population, both sexes, at the same ages, this latter great disparity being, no doubt, largely due to the casualties among women during the child-bearing period.

As the wear and tear of practice begins to tell this advantage is soon lost, so that during the period from 30 to 70 the death rate of physicians is 8 per cent. greater than that of all males, and during the period from 40 to 70 it is more than 11 per cent. greater than that of both sexes.

An examination of the causes of death in the following table reveals the result of the exposure, irregular hours, broken rest, and mental anxiety, which are the lot of the average practitioner :

TABLE III.—*Principal causes of deaths of physicians in Illinois during the ten years ended December 31, 1887*

| Causes. | Number. | Per Cent. of Total. |
|---|---|---|
| 1. Phthisis | 122 | 15.3 |
| 2. Diseases of the Lungs and Respiratory Apparatus | 115 | 14.4 |
| 3. Diseases of the Brain and Nervous System | 105 | 13.1 |
| 4. Zymotic Diseases | 82 | 10.0 |
| 5. Misadventure and Violence | 75 | 9.4 |
| 6. Diseases of the Heart and Blood-vessels | 67 | 8.4 |
| 7. Diseases of the Stomach and Digestive Tract | 48 | 6.0 |
| 8. Bright's Disease | 31 | 3.8 |
| 9. Diseases of the Liver and Appendages | 26 | 3.2 |
| 10. Enteric Fever | 24 | 3.0 |

Of the grouped causes of death above given it will be seen that consumption, diseases of the respiratory organs (including 91 from pneumonia), and Bright's disease caused 268 deaths, or more than one-fourth of the total. If to these be added a share of the deaths from diseases of the heart—the sequelæ of rheumatism—a fair estimate may be made of the effect of exposure to the vicissitudes of weather upon the wear and tear of medical life. As a result of mental strain and anxiety, of insufficient, irregular, and interrupted sleep, and similar causes is the total of deaths from diseases of the brain and nervous system, embracing forty-three from various forms of paralysis. In the group of zymotic diseases (enteric fever given separately) there were five deaths from diphtheria, one each from small-pox and yellow-fever, and eight from traumatic infection (septicæmia, etc.), all contracted from attendance upon patients.

Less creditable to the morale of the profession are the eighteen deaths from over-doses of opiates and narcotics, the seven admitted suicides, and the deaths from alcoholism, direct and indirect—twelve of the former and at least eight of the latter. There is this to be said, however, in this connection, that the proportion of mortality from these causes is steadily diminishing, and my observation shows that this diminution is largely the result of an amelioration of the conditions, especially of country practice, due to better roads and methods of locomotion, increased comfort in living, and less physical strain upon the practitioner. Ten years ago the resort to stimulants upon exposure to the weather and under the harsher conditions of practice which then obtained was much more common than it is to-day. And this is also true of the use of opiates and hypnotics. The practitioner—familiar with their power to temporarily stimulate to further endurance, or to procure sleep when nervous and exhausted—had formerly greater temptation to resort to the use of these agents, always ready to hand.

While there is a total of twelve deaths reported during the ten years as due to "alcoholism" direct, there has been only one in the last four years; and of the eighteen deaths from "over-doses of opiates and hypnotics" in the entire period, there has been only one in the last three years. In addition to amelioration in the conditions of practice as a cause of this result, it is only fair to take into consideration, also, the improved moral status of the profession in this State.

Although the figures and deductions here submitted are believed to be substantially accurate—being, if anything, understatements—they are offered only as a provisional contribution to the study of the subject, which is by no means exhausted. The numbers under observation and the period covered are greater than anything heretofore utilized for this purpose in this country, so far as I am aware, and have cost much labor, which may be materially lightened in the future by very little effort on the part of physicians in making returns of death certificates, and by county clerks in forwarding them to the office of the board. It is hoped that the interest which this presentation of the subject may reasonably be expected to arouse will lead to this result.

MEDICAL-PRACTICE ACT.

There were issued during the quarter 138 certificates authorizing the practice of medicine in the State—126 of which were to graduates upon diplomas from medical colleges in good standing; eleven to non-graduates upon proof of ten or more years of practice in the State prior to July 1, 1887, and one upon satisfactory examination; six duplicates were also issued upon proof of the destruction of the originals. Certificates were issued to twenty-six midwives—fifteen upon diplomas or licenses, and eleven upon examination.

Of the 149 applications for medical-practice certificates, eleven were refused to applicants who were unable to comply with the provisions of the law and the requirements of the board based thereon. There were also eight applicants who presented diplomas from colleges which do not fully comply with the prescribed standard of study and instruction. Certificates were issued to these only after passing examinations in the neglected branches, or furnishing evidence of the necessary proficiency in the omitted qualifications. Two applications have been received for itinerant license, one from Michigan and one from Indiana. In accordance with the action of the board heretofore, they were refused. Since July 1, 1887, $9,700 have been refused by the board for licenses of this character.

MEDICAL EDUCATION.

More interest is now evinced in the subject of medical education than ever before. This is true of the country generally, and during the quarter, communications have been received from both extremes of the continent showing the progress being made. The Secretary of the Maryland State Board of Health announces the enactment by the Maryland legislature of a medical-practice act similar to that of Illinois. The Secretary of the California State Board of Medical Examiners gives notice that after the 1st of April, 1891, no diploma issued thereafter will be recognized as the basis of a certificate entitling to practice, unless the college shall have exacted three full years of study, and attendance upon not less than three full regular courses of lectures, delivered during three separate years, as conditions of graduation. The St. Louis Medical College, whose course of instruction heretofore has been thorough, has decided to lengthen its lecture term to six months. The Medical Department of the University of Wooster, at Cleveland, Ohio, and the Medical College of Ohio, at Cincinnati, will, at the next session, require attendance upon a three years' graded course of lectures.

IM MEMORIAM.

The Secretary was instructed to transmit a message of sympathy to the widow of Dr. Cornelius Rea Agnew, and to prepare an expression of the sentiments of the board upon the death of that distinguished physician, sanitarian, and medical teacher.

The secretary spoke of the cordial support and ready interest Dr. Agnew had always given to the efforts of the board for the elevation of the status of the medical profession and the promotion of practical sanitary measures.

### ON THE TREATMENT OF PHTHISIS BY CREOSOTE.

[Translated and Abstracted by Dr. Lester Curtis.]

Two papers on this subject have recently appeared in the *Berliner Klinische Wochenschrift*, one by Dr. Brun, in No. 8, and one by Dr. Peter Kaatzer, in No. 11.

Dr. Brun has used the creosote during the last eight years in 1,700 cases of phthisis, some very bad, with remarkably good results. A sample case is as follows:

"Mr. S., chemist from S., came under my treatment July, 1886. He is thirty years of age. Originally he was a large strong man. He comes of a healthy family, and was well until November, 1885, when he became ill with a cough, expectoration, and the well-known phenomena of hectic. As a result his weight diminished some forty pounds. Patient appeared very much emaciated, was very anæmic, with sharply defined red spots on the cheeks pulse 120 to 130 per minute. Continued fever, morning 38.4° (101.12° Fahr.), evening, 39.5° to .7 (103.1° to .5 Fahr.); night sweats; loss of appetite; inclination to diarrhœa. In the sputum are masses of bacilli. A local cavity the size of the fist and firm infiltration of the whole left upper lobe; right, slight dullness above and limited crepitant râle in the upper part of the apex. The prognosis seemed gloomy, and more that something might be done, creosote was given, on account of the existing diarrhœa, in pill form with opium.

"Almost contrary to expectation the appearance changed; the diarrhœa disappeared, the digestion became normal, appetite appeared, the nutrition improved, and the respiratory symptoms disappeared. Then the stronger form of creosote wine was ordered. It was borne, and was used during the treatment here for two months. At the end of this time the weight of the body had increased about fifteen pounds. The fever had lost its continued character, the temperature was normal in the morning, at evening it was 38.2° to 38.5° (100.8° to 101.3° Fahr.). Expectoration and cough had remarkably lessened. In the sputum bacilli appeared only rarely, and the board-like dullness had considerably cleared up, the cavity decreased, the secretion within it diminished.

"Patient left Lippspringe with instructions to continue the use of the creosote wine during the winter, and in the spring return here. This occurred, and the reports received every month continued favorable. Some weeks after the return home the fever entirely disappeared without further assistance and the patient became so strong that he was able to resume his duties as chemist of a sugar manufactory.

"In June, 1887, he came back and exhibited a picture of health. The body-weight was greater than before the illness, cough and expectoration only in the morning, in a minimum degree, no dyspnœa, bacilli entirely gone. Signs of a cavity could no longer be detected within the boundary of contraction of the left apex. It was difficult to awake, in the vigorous man, the consciousness of the necessity of further precautions."

A second case is given of a patient from a tuberculous ancestry, who had for many years suffered from catarrh of the respiratory organs. After a pneumonia, gangrene occurred in the lungs, with the usual constitutional disturbances. The creosote was given, and was followed by restoration to a condition quite as good as that before his illness.

The maximum dose of creosote which Dr. Brun uses is from 0.4 to 0.45 in a day. He insists on the necessity of using that prepared from beech tar.* The improvement corresponds with the amount which the patient is able to take. The form which he prefers is creosote wine, his formula being "Creosote fagen 13.0, T. A. gentian 30, spirit. vin. rectif. 250, vin. Tokayens q. s. ad. colat. 1000. S.—One tablespoonful to be taken three times daily, diluted with water."

The medicine is usually well borne, the worst thing about it being the pungent, disagreeable taste. Patients, however, usually soon become accustomed to this and do not mind it.

He has also used the "Ruess-Sommerbrodt's" capsules of tolu balsam 0.2, and creosote 0.05, but does not like them so well on account of their liability to derange the stomach.

When there is tendency to diarrhœa he uses the creosote in pill form with opium, "Creosote fag. 2 to 2.5, op. 0.3 to 0.4: 50. S.—One pill to be taken every three hours." With this treatment the diarrhœa usually stops promptly. Then he begins with the creosote wine.

Along with this treatment he usually employs creosote inhalations. For this pur-

* It should be borne in mind that ordinary commercial creosote is only carbolic acid, probably impure, and is not fit for internal use.—L. C.

pose he recommends Feldbausch's nasal inspirator. "This consists of two small tubes one and a half to two centimetres long, designed for each nostril. These are connected by a semi-circular middle piece. The interior contains filter paper upon which the creosote is dropped. While in use the tubes are placed entirely within the nasal canal so as to be scarcely perceptible to a second person. The irritation of the interior of the nasal cavity by the application is insignificant, and after several introductions is scarcely perceptible, so that the little apparatus may be used during any sort of occupation by day, and even throughout the night."

Dr. Kaatzer has treated somewhat more than a hundred and sixty cases with creosote. He considers that more than 10 per cent. of these have perfectly recovered. He has made repeated examinations of the sputum in each of these cases, at considerable intervals, without finding any bacilli or remains of elastic tissue.

"In 40 per cent. of my patients there occurs, during long treatment by creosote, if not recovery, still, such improvement that they are enabled to resume their occupations, which had been given up, and could work again with a feeling of renewed vigor. Among these is worth mentioning a bookkeeper. Along with bacillary lung phthisis, he suffered with bacillary otitis, on account of which a well-known authority was consulted. The patient mentioned came under my treatment in the summer of 1885. According to the account of the before-mentioned university teacher, no case of this affection that had come under his observation had lived more than a year. The unfavorable prognosis was not fulfilled. On the other hand, the number of bacilli in the sputum in the summer of 1887 was, by repeated investigation, very scanty—an average of one bacillus in several fields of the microscope. Patient had, besides, with the exception of a nine weeks' furlough, which he has spent every year in Bad Rehburg under my immediate supervision, fulfilled perfectly the trying duties of his occupation."

The formula used by Dr. Kaatzer is:

R.

| | |
|---|---|
| Creosote puressimi e fag....... | 2.0 |
| Spts. vini rectif................ | 30.0 |
| Tr. gentian.................... | |
| Ext. coffea ana................ | 10.0 |
| Aq. dest...................... | 100.0 |

S.—To be well shaken. A tablespoonful three times a day in half a cup of milk."

He is not sure but it would be better to take the same daily quantity in smaller doses from four to six times a day rather than the larger doses three times a day.

---

### ALANINATE OF MERCURY; A NEW ANTISYPHILITIC.

Schmiedeberg's suggestion, in his *Compendium der Pharmacologie*, that combinations of mercuric oxide, with certain amidic acids of the fatty series (acetamidine, glycocollamine, as paragine) should act well in the treatment of syphillis, and the fact that these preparations have the advantage of being very soluble and easily absorbed, induced Dr. R. De Luca, assistant in the clinic of Professor Ferrari in the University of Catania, to make some experiments with mercuric alanine in the treatment of syphilis, and these he has now published. Preliminary experiments on animals (rabbits, Guinea-pigs, and dogs) showed that the drug was not very poisonous.

The alaninate of mercury used was prepared in the following manner: One part of alanine was dissolved in twenty parts of distilled water, which was then gradually raised to the boiling point; while the liquid was boiling it was poured over a small quantity of mercuric oxide (HgO) until it was all dissolved. Then the liquid was filtered and evaporated, and the residue crystalized, and a whitish substance, mercuric alanine, was obtained, which under the microscope showed the characteristic needle-shaped crystals grouped in crosses and tufts. When thus prepared alaninate of mercury is soluble in three volumes of cold distilled water; this aqueous solution is perfectly colorless, clear, is changed neither by exposure to the air nor to light, and will keep indefinitely. In dilute solution it will not coagulate albumen; in concentrated solution its coagulating action is limited to its causing a cloudiness of that part of the liquid with which it comes immediately in contact. In other respects it has the general properties of other salts of mercury.

On his patients De Luca used the drug in three different solutions of 4, 8, and 10 milligrammes, to 1 ccm. of distilled water, (gr. 1-16, 1-8, and 1-6, to 16 m.), both for internal use and for injections, subcutaneous or intermuscular. The drug was used by injection in twenty cases; nineteen cured and one improved. The daily quantity used on each patient (all adults) was from five to ten milligrammes. The average number of days of treatment for each patient was 37.05; average number of injections to each patient, 27.7; average age of patients, 23.2 years. In the case that improved but was not cured, the patient stopped treatment. All the cases were those of secondary syphilitic lesions. Each patient received an average quantity of 228 milligrammes (gr. 3.5). Only three times did suppuration occur at the site of injection. As regards the duration of treatment, then, it is seen that mercuric alanine has some advantage over the bichloride; as to the quantity to be used it has a decided advantage, the quantities being as 22.8 to 42. With regard to the efficacy of the alaninate, in twelve cases (out of the twenty) that could be seen to ascertain if any recurrence had taken place, there was only one case of recurrence, in a case of syphilitic papule of the larynx. From this it seems that the alininate gives more permanent results than other mercurial preparations. Further, in not a single case in which the drug was used by injection was there any stomatitis or other unpleasant effect produced.

From a tabulated statement of twenty cases treated by the internal use of the drug, it is seen that for the cure of ten adults with secondary syphilitic lesions, there was an average of 45.4 days and 641 milligrammes to each patient. Of ten children treated one died on the third day. For the cure of the remaining nine, of an average age of 7.4 months, with hereditary or acquired syphilis, each required an average of 54.6 days, and 159 milligrammes of the drug. Of the nine cured six were seen in from eight to nine months after treatment was stopped, and there was no recurrence in any one them. There was not a case of stomatitis, and only one case of intolerance of the drug, which was completely controlled by the administration of cocaine. Regarding the internal use of the alaninate, then, it is not to be preferred to the tannate or the phenate of mercury in the opinion of DeLuca, but the easy tolerance of the drug, and the excellent manner in which it acts on syphilis in infants, make it an important addition to the list of antisyphilitics. The child that died was only two months old, and was in a desperate condition when it came under treatment. DeLuca claims that the decidedly calmative effect of the drug gives especially happy results in children.

The author thinks that the drug is worthy of confidence, and that time may prove that it will give good results in the late manifestations of syphilis.—*La Riforma Medica*, March 22 and 23, 1888.

### THE EXCISION AND SCRAPING OF CARBUNCLE.

Mr. Rushton Parker, of Liverpool, says that there are few procedures in practical surgery that better deserve recommendation than the local extirpation of carbuncle, of which the pain and tenderness are thereby ended, and the state of gangrene, always virulent and sometimes inveterately spreading, replaced by simple inflammation in a healing sore. If the carbuncle be small enough, or seen early enough, an abortive treatment may be sometimes practicable by syringing through it an effective antiseptic liquid; but we seldom get such a good chance of doing this in carbuncle as in boil. He has several times arrested a boil in its early commencement, when little more than a pustule in a hair-follicle, by injecting strong carbolic or sublimate solution a few times, at short intervals, with a hypodermic syringe. But the manipulation necessary for this, even in a small carbuncle, is at least as painful and as prolonged as the excision of the virulent centre (or centres), and not appreciably more saving of tissue. The effect of excision is an immediate end to the very acute painfulness, and a gradual but sure end to the constitutional disturbance. The carbuncle may require erasion or excision, or both, according to the density of the inflamed tissues. Mr. Parker gives five cases, some of which may be quoted here:

CASE 1.—Girl, aged 19 years, with an acute brawny inflammation of the left side of the chin, of four days' duration. Under chloroform an incision was made, and the nodule inspected within, as there was a question of malignant pustule. The tissues in the centre were gray and purple, and the nodule was decided to be a carbuncle. A conical piece was excised, pure carbolic acid put into the wound, with a sublimate dressing laid on. The inflammation melted rapidly away, day by day, and the girl was entirely well in a fortnight. Here the best practicable form of immediate local extirpation seemed to be excision with a sharp knife, on account of the density of the tissues. The virulent centre was about the size of a pea, yet the collateral swelling was as big as half a walnut.

CASE 2.—A night-soil carter, aged 23, had a pimple on his neck. This he picked, and on the second day after his neck began to swell, and his head to ache intensely. On the fourth day he came to the Liverpool Royal Infirmary, with the left side of his neck in a swollen, red, angry state, very much like malignant pustule; his temperature was 104°. Excision of the centre, dirty-gray and purplish, with some surrounding skin, was done without an anæsthetic shortly after his admission. Pure carbolic acid was put in, and sublimate dressings applied. Temperature 105° in the evening, but fell to 100° during the night. Temperature remained above 100° until the third day after admission. On clinical grounds the case was regarded as a mild form of malignant pustule, but as neither the juice of the excised fragment nor the patient's blood nor urine showed bacilli under the microscope, the case is now considered to have been one of carbuncle with unusually severe symptoms, presumably due to ptomaine poisoning.

In a case of carbuncle on the back of the neck, a crucial incision was made under chloroform, and most of the carbuncular tissue being soft and pulpy, it was removed by means of the sharp spoon. The bleeding was abundant, but was controlled with hot water and pressure with sponges. The patient became perfectly comfortable, and made a good recovery under dressings of sublimate and eucalyptus ointment.

The use of pure carbolic acid has the advantage that the acid produces local anæsthesia in addition to its antiseptic qualities—*British Medical Journal*, March 31, 1888.

---

### THE PRESENT STATE OF CARDIAC THERAPEUTICS.

Dr. JAMES STEWART has an excellent article on this subject in the *Canada Medical and Surgical Journal*, February, 1888. In speaking of the treatment of acute inflammations of the endocardium he points out the great value of rest, which means low blood-pressure, and consequently less work for the valves to do. The rest may be aided by drugs that lower blood-pressure, such as chloral. He does not approve of blood-letting and blisters over the præcordia, because the latter is injurious, and the former of only temporary benefit. Of the treatment of heart diseases during the period of compensation he speaks favorably of Oertel's plan, which consists in strengthening the muscular system, including the heart, by regular and graduated outdoor exercise; aiding nutrition by a diet rich in albúmen, and by careful regulation of the quantity of fluids taken. This plan is probably adapted to cases of threatened cardiac failure from beginning fatty degeneration and from chest deformity or pulmonary disease. When there is such loss of compensation as to render exercise impossible, then digitalis is of great value. The first marked effect of cardiac failure is diminution of the aortic blood-pressure, which is shown by a diminished excretion of urea. It is safe to continue the administration of digitalis so long as it causes an increase in the quantity of urine excreted. The diuretic power of digitalis is dependent entirely on its power of raising an abnormally low blood-pressure, and in order to do this it must be given in full doses. If, after being increased by digitalis, the urine diminishes considerably, the drug should be discontinued immediately; and this decrease is a warning that should never be neglected. There is a great difference in the quantity of digitalis necessary to cause diuresis, since patients differ greatly in their susceptibility to it. In the majority of cases 40 minims of the tincture, given four times a day for three days, will cause the diminution, but sometimes so much as half an ounce in divided doses is required.

The best results are obtained in these cases by absolute rest in bed, with full doses of digitalis. Scillain, helleborein, eleandrin, adonidin, convallaramin, and strophanthin also increase the blood-pressure and slow the heart. Mercury is a direct diuretic; hence the value of the old combination of digitalis, squills, and mercury. Dr. Stewart speaks hopefully of strophanthin, and favorably of the effect of caffeine, especially of the natro-salicylate of caffeine, which is a powerful direct diuretic acting on the epithelium of the convoluted tubules, and probably on that of the glomeruli. It acts promptly. Good results may be had from a combination of digitalis and caffeine.

---

### WOUND OF THE LEFT VENTRICLE; RECOVERY.

Dr. A. P. Kiawkoff reports the remarkable case of a Cossack, who received a wound from a dagger in the left side of his chest. A physician saw the patient almost immediately, and found him lying on the ground, unconscious, and with stertorous breathing. The man's chest was covered with blood, and in the fourth intercostal space, in the mammillary line, and parallel with the border of the ribs, was a wound about 1.5 inches long from which blood was still flowing. The wound was cleansed, a compress put on, and restoratives applied, after which the patient became conscious. On the next day his general condition was very good; pulse 90 and small, temperature 37.8 C. Examination of the chest showed the upper boundary of cardiac dullness in the fourth intercostal space, and the cardiac impulse was faulty. The lower percussion boundary was at the upper border of the seventh rib. The right boundary reached beyond the right parasternal line, and the left boundary about three centimetres beyond the left mammillary line.

On the third day the patient was taken to a hospital, which he left after a stay of four weeks, cured. Five days after leaving the hospital he suddenly dropped dead while trying to lift a heavy object.

The autopsy showed that the heart wound was completely closed, and the outer layer of the pericardium adherent to the wall of the thorax. The pericardium was filled with dark blood; in the left ventricle was a gaping wound, 1.4 centimetres long, the edges of which were thickened, and the neighboring muscular tissue in a state of fatty degeneration; there was also acute endocarditis.

The case, then, was one of healed wound of the left ventricle, but the patient died from too early strain on his weakened heart.

The cicatrix was too young, and the endocarditis had not passed off. The heart could not stand the strain of the sudden inrush of blood, and the cicatrix ruptured. This case makes 7 per cent. of recoveries of recorded cases of heart wound.—*Centralblatt für Chirurgie*, No. 12, 1888.

### CYSTIC TUMOR OF THE LARYNX.

Dr. C. M. Desvernine, of Havana, reports a case of this rare affection. It was the case of a man, aged 70 years, a Cuban, who came under Desvernine's treatment for chronic hoarseness and symptoms referable to the digestive organs; intense epigastric pain, paroxysmal, and regurgitation of food within a few minutes after eating. The man knew nothing of his family history, but he was neither alcoholic nor syphilitic. He had dysphagia from organic (cancerous) stricture of the œsophagus at its lower third, and there was typical bilateral dysphonia. Laryngoscopic examination showed that the organ was normal in all its parts except the right vocal cord, the middle third of which was so enlarged as to present a general fusiform appearance. Its bilateral movements were correctly performed, abduction and adduction being full, complete, and energetic. General tension, on the contrary, was *nil.* During efforts at vocalization the appearance of the cord was not changed, and during the utterance of liquid sounds the corresponding arytenoid was dragged and forcibly inclined towards the centre of the larynx. The color of the cord was a faded white, reflecting less light than the other. It was most probable that the cord was invaded by a dense and inelastic product, and the diagnosis of interstitial fibroma and diffuse chorditis was made.

The patient died of the carcinomatous infiltration of the œsophagus. Direct laryngeal inspection showed the same appearance as the ante-mortem examination. The fusiform enlargement occupied the anterior part of the middle third of the cord, and measured 6 mm. in its greatest diameter. It fluctuated on pressure, and a few drops of a clear mucoid liquid escaped. In the cord was a cavity measuring 5 mm. in transverse diameter, and 3.5 mm. vertically.

There is up to this time, says the author, no complete histological record of such a tumor, and he promises the results of the histological examination of this case at an early date.—*Cronica Mêdico-Quirúrgica de la Habana*, March, 1888.

### HAIR-TUMOR OF THE STOMACH; GASTROTOMY; RECOVERY.

John Berg reports the case of a married woman, age 26, who had suffered for three years with symptoms of dyspepsia and anæmia, and with attacks of vomiting of glairy mucus. For two years a tumor had been noticed in the epigastric region, and it had grown more rapidly during the past six months. She entered the Seraphim Hospital in Stockholm on May 31, 1887. In the epigastric and left hypochondriac regions, between the prolongation of the parasternal line and the left mammillary line was a tumor as large as the hand, with a concave upper and a convex lower border. It could not be displaced towards the region of the kidney. The spleen was in its normal position. Laparotomy showed that the tumor was in the stomach, which was opened by an incision six or eight centimetres long, parallel to the greater curvature. The tumor was composed of hair, short and long, forcibly compressed. It was cut up and removed piecemeal. The tumor weighed about 900 grammes. The wound was closed by 23 sutures, in a double row. Union occurred by first intention, and complete recovery ensued. The mother of the woman said that when she was about three years old she was in the habit of chewing and swallowing hair, but the patient denied having done so since she could remember. With the cases of Schonborn and Knowsley Thornton, this makes three cases of this operation, all successful, for hair-tumor.—*Nordiskt Medicinskt Arkiv*, Bd. 19, No. 25.

### CALOMEL IN PHAGEDÆNA.

Surgeon-Major T. J. GALLWEY, at Newcastle, Jamaica, writes to the *British Medical Journal*, of March 31, 1888:

I had a case of phagedænic ulceration of the under surface of the glans penis under my charge last August, which defied the recognized treatments of this disease. I applied nitric acid in the most thorough manner on six different occasions during a period of eighteen days without success. I then applied pure carbolic acid, but the disease again returned. Constitutional treatment with opium was adopted throughout. For six days the patient sat in a hot-water hip-bath on an average about four hours daily, without any appreciable effect on the course of the disease. The condition of the penis on the twenty-first day was as follows:

A large ulcer existed, covering the entire under surface of the glans, molding it like the mouth-piece of a flute, and extending to the reflected foreskin in the vicinity of the ulcer. A third of the glans had been destroyed. The surface of the ulcer was covered with a reddish grey secretion, irregularly disposed, and pierced here and there by large red granulations. The edges were angry and undermined.

I applied calomel powder on the twenty-first day of the disease, spreading it thickly, and pressing it well into the interstices of the ulcer. The calomel acted like magic; the ulcer began to heal rapidly. Now and then a suspicious spot appeared, but it was at once dissipated by a thorough application of the calomel. The patient made an excellent recovery, and was very pleased at the result, for he believed he was going to lose the whole affair. I was tempted to use calomel, as I had found it very useful in all forms of syphilitic ulceration.

### THE VALUE OF IODOL.

Dr. ASSAKY has made a number of experiments to determine the value of this compound. It should be a yellow-brownish powder, inodorous when recently prepared, almost insoluble in water, soluble in alcohol, ether, fatty oils, and crystallizable acetic acid, and should contain from 85 to 89 per centum of iodine.

In Assaky's hands, operation wounds that were dusted over with iodol healed by primary union. In sloughing and suppurating wounds it proved an excellent antiseptic, rapidly drying up all purulent secretion. Since it is not toxic it may be used in large quantity without inconvenience. Assaky claims that it is highly probable that it destroys pyogenic organisms. It gives excellent results when used in and on wounds having a tendency to ulcerate, and transforms them in a short time into freshly granulating surfaces. While it is a good dressing for indurated chancres, it gives variable results when used on soft chancres.

When taken internally, in doses of from 40 centigrammes to 2 grammes a day, it does not cause functional troubles, even when continued for a long time. It causes slight congestion of the nasal and conjunctival mucous membranes, but this disappears when large doses are taken. It does not cause albuminuria, but, on the contrary, it has perhaps a curative action in some cases of albuminuria. It has given good results in tertiary syphilitic affections, and in surgical scrofuloses, acting more rapidly than the alkaline iodides.

It may be used in powder, in a glycero-alcoholic solution, in gauze or iodolated collodion, in ethereal solution, or it may be incorporated with vaseline or lanolin.—*L'Union Médicale*, March 18, 1888.

### MUSHROOM-POISONING.

Dr. Matthes, in the *Berliner Klinische Wochenschrift*, No. 6, reports five cases of poisoning by mushrooms, including one nursing child. In all these cases the symptoms were alike. When first seen the patients were delirious, as though from alchohol. The face was cold and pale, lips bluish, respiration superficial and very frequent, pulse slow and interrupted, pupils wide and without reaction to light. The treatment was emetics and purgatives and hypodermic injections of ether.

Soon tonic spasms occurred, which were made specially noticeable by the ether-injections. They also occurred on shaking the bed, or by throwing a bright light in the face, and, even, spontaneously. The convulsions were repeated at intervals of eight to ten minutes, and lasted about two hours. At one time there appeared to be danger of heart failure, and the doctor contemplated hypodermic injections of strychnine, but was deterred by the fear of complaints, in case of a fatal result.

Pieces of the common fly mushroom (*Amanta rubesens*), along with other edible mushrooms, were found in the vomited matter.

The first symptoms occurred three and a half to four hours after eating. They were described by one of the patients as severe gastralgia, followed by symptoms like alcoholic intoxication. The next morning the patients were in a fair condition, only suffering with a disagreeable feeling in the head.

The special interest in the case is the convulsions, which are not common.

LESTER CURTIS.

### MENSTRUATION FROM A LAPAROTOMY SCAR.

Professor GEORGE E. REIN recently exhibited to the Obstetrical and Gynæcological Society of Kew a menstruating woman, upon whom he operated about three years ago for a large cyst of the right ovary, the pedicle being fixed in the abdominal wound. The patient made a good recovery, and the wound healed entirely except that at one part of the scar there remained a very small slough, which fell off just before the occurrence of menstruation, after which there was a constant flow of blood from the denuded surface during the whole of the menstrual period. This had occurred regularly every month since the woman recovered, the scar usually beginning to bleed a little earlier than the uterine flow appears. The blood from the cicatrix has the characteristic menstrual odor. It is possible that a Fallopian tube or one of the cornua of the uterus was stitched into the abdominal wound with the pedicle. As Professor Rein will have to perform a second operation soon for disease of the left ovary, he hopes to discover the cause of this rare and very interesting occurrence.—*Vratch*, February 18, 1888.

### SEPTICÆMIA AND PUERPERAL THROMBOSIS.

FATTY HEART; TREATMENT BY MASSAGE, SPARTEINE, AND EXERCISE; RECOVERY.

Dr. D. ANTONIO PEÑAFIEL, of Mexico, reports an interesting case under the above title. The massage was applied to the œdema caused by the thrombosis. He believes massage and mechanical exercise was a very important part of his treatment. The sparteine was given in daily doses of from one milligramme to one centigramme, and it had an almost immediate effect upon the heart. It increased the quantity of urine without causing the inconveniences seen in the administration of other diuretics, and without showing any cumulative action. The diet in this case was almost exclusively of milk. The case was not one of classic Stokes' fatty degeneration, the diminution of cardiac force being neither permanent nor uniform. There were no cerebral symptoms, and no disturbance of respiration in consequence of left ventricular weakness. The heart was rather in a state of obesity than of fatty degeneration.—*Gaceta de Mēdica de Mexico*, Tom. 23, Ent. 5.

### TRANSPLANTATION OF THE CORNEA.

VON HIPPEL reports two cases of successful transplantation of the cornea of the Guinea-pig, in leucoma, in which Descemet's membrane was not involved. In one case the central leucoma was removed by means of a specially constructed trephine of a diameter of about four mm., up to the membrane of Descemet. A piece of the cornea of a Guinea-pig the size of the piece removed from the human eye was transplanted. This healed readily and remained transparent. One of the patients who could before the operation only count fingers a two metres, regained sight up to 20-200. The second case was one in which a central leucoma remained after ulcus serpens. In this case a piece of cornea four mm. in diameter was transplanted in the same manner, and with equally good results.—*Centralbl. für die medicinischen Wissenchaften*, January 28, 1888.

### NEW METHOD OF REDUCING STRANGULATED INGUINAL HERNIA.

G. S. PERRO reports a series of successes with the following method:

After the pelvis has been raised on a pillow, and the thigh flexed and abducted, the operator grasps the scrotum and the hernial tumor, bends it slightly over and against the wall of the abdomen, and presses upon it in such a manner that the index finger of the right hand is carried into the inguinal canal, and in the direction of the horizontal ramus of the pubis by a turning and boring motion. In a short time the strangulated part slips back into the abdomen, and the other part of the hernia follows. By this method Perro has succeeded in reducing six cases of strangulated hernia after his colleagues had spent from twelve to thirty hours in vain attempts at reduction.—*Centralblatt für Chirurgie*, November 12, 1888.

